# Estimated US Pediatric Hospitalizations and School Absenteeism Associated With Accelerated COVID-19 Bivalent Booster Vaccination

**DOI:** 10.1001/jamanetworkopen.2023.13586

**Published:** 2023-05-19

**Authors:** Meagan C. Fitzpatrick, Seyed M. Moghadas, Thomas N. Vilches, Arnav Shah, Abhishek Pandey, Alison P. Galvani

**Affiliations:** 1 Center for Vaccine Development and Global Health, University of Maryland School of Medicine, Baltimore; 2 Center for Infectious Disease Modeling and Analysis, Yale School of Public Health, New Haven, Connecticut; 3 Agent-Based Modelling, York University, Toronto, Ontario, Canada; 4 The Commonwealth Fund, New York, New York

## Abstract

**Importance:**

Adverse outcomes of COVID-19 in the pediatric population include disease and hospitalization, leading to school absenteeism. Booster vaccination for eligible individuals across all ages may promote health and school attendance.

**Objective:**

To assess whether accelerating COVID-19 bivalent booster vaccination uptake across the general population would be associated with reduced pediatric hospitalizations and school absenteeism.

**Design, Setting, and Participants:**

In this decision analytical model, a simulation model of COVID-19 transmission was fitted to reported incidence data from October 1, 2020, to September 30, 2022, with outcomes simulated from October 1, 2022, to March 31, 2023. The transmission model included the entire age-stratified US population, and the outcome model included children younger than 18 years.

**Interventions:**

Simulated scenarios of accelerated bivalent COVID-19 booster campaigns to achieve uptake that was either one-half of or similar to the age-specific uptake observed for 2020 to 2021 seasonal influenza vaccination in the eligible population across all age groups.

**Main Outcomes and Measures:**

The main outcomes were estimated hospitalizations, intensive care unit admissions, and isolation days of symptomatic infection averted among children aged 0 to 17 years and estimated days of school absenteeism averted among children aged 5 to 17 years under the accelerated bivalent booster campaign simulated scenarios.

**Results:**

Among children aged 5 to 17 years, a COVID-19 bivalent booster campaign achieving age-specific coverage similar to influenza vaccination could have averted an estimated 5 448 694 (95% credible interval [CrI], 4 936 933-5 957 507) days of school absenteeism due to COVID-19 illness. In addition, the booster campaign could have prevented an estimated 10 019 (95% CrI, 8756-11 278) hospitalizations among the pediatric population aged 0 to 17 years, of which 2645 (95% CrI, 2152-3147) were estimated to require intensive care. A less ambitious booster campaign with only 50% of the age-specific uptake of influenza vaccination among eligible individuals could have averted an estimated 2 875 926 (95% CrI, 2 524 351-3 332 783) days of school absenteeism among children aged 5 to 17 years and an estimated 5791 (95% CrI, 4391-6932) hospitalizations among children aged 0 to 17 years, of which 1397 (95% CrI, 846-1948) were estimated to require intensive care.

**Conclusions and Relevance:**

In this decision analytical model, increased uptake of bivalent booster vaccination among eligible age groups was associated with decreased hospitalizations and school absenteeism in the pediatric population. These findings suggest that although COVID-19 prevention strategies often focus on older populations, the benefits of booster campaigns for children may be substantial.

## Key Points

**Question:**

Would accelerated COVID-19 bivalent booster vaccination uptake in the US be associated with decreased outcomes of pediatric hospitalizations and student absenteeism?

**Findings:**

In this decision analytical model using US population estimates, a simulation model revealed that booster campaigns achieving an uptake similar to seasonal influenza vaccination could have prevented an estimated 10 019 pediatric hospitalizations and 5 448 694 days of school absenteeism from October 1, 2022, to March 31, 2023.

**Meaning:**

These findings suggest that although COVID-19 prevention strategies often focus on older populations, the benefits of booster campaigns for children may be substantial.

## Teaser

This decision analytical model estimates whether increases in COVID-19 bivalent booster vaccination uptake in the eligible US population would be associated with reduced hospitalizations and school absenteeism among children.

## Introduction

COVID-19 substantially disrupted many elements of normal life, with notable consequences for children. As of March 6, 2023, the pandemic had been responsible for more than 185 000 hospitalizations and 1700 deaths in the pediatric population.^1,2^ Among interventions implemented, school closure was the most consistently applied measure globally in 2020, affecting more than 90% of students worldwide.^3^ In subsequent academic years, students have faced challenges including illness-related absenteeism, pandemic-related instability in their personal lives, outbreak-related school closures, and transition to virtual or hybrid learning formats. A consequence of these experiences has been lower test scores among children receiving education during the COVID-19 pandemic compared with their prepandemic counterparts.^4^ Preventing severe illness among children and maintaining high levels of school attendance should be important goals for policy makers.

Vaccines are among the most important tools available to prevent COVID-19. While waning protection and immune escape have posed substantial challenges to pandemic control,^5^ a bivalent COVID-19 booster vaccine is available that specifically targets some of the highly transmissible Omicron subvariants.^6^ We hypothesized that COVID-19 vaccination campaigns achieving high booster coverage among eligible individuals could have markedly reduced severe disease and deaths across all ages and prevented school absenteeism and consequent educational disruption. Using a simulation model, we evaluated whether benefits would have accrued to the pediatric population by improving bivalent booster coverage among both children and adults. We quantified the associations of the booster campaign with hospitalization, pediatric isolation days, and school absenteeism.

## Methods

### Ethics

For this decision analytical model, we used publicly available data for reported cases of COVID-19, hospitalizations, and deaths^7^ (eMethods in Supplement 1). Because data were not collected specifically for this study and no identifiable personal data were used, specific ethical approval and informed consent were not required in accordance with York University research ethics guidelines for program evaluation activities relying on secondary use of anonymous data. This study followed the Consolidated Health Economic Evaluation Reporting Standards (CHEERS) reporting guideline for decision analytical models and simulated modeling studies.^8^

### Study Design and Population

We used a simulation model to estimate COVID-19 health outcomes for the US population under a range of bivalent booster uptake scenarios from October 1, 2022, to March 31, 2023. These scenarios included a baseline scenario matching daily COVID-19 bivalent booster vaccination rates and 2 counterfactual scenarios comprising a bivalent booster with (1) a campaign that achieved coverage equivalent to one-half of the age-specific influenza vaccine uptake in the 2020 to 2021 season^9^ and (2) a campaign that mimicked the same age-specific influenza vaccine coverage for eligible individuals aged 5 years or older by September 30, 2022. Outside the 3-month duration of the counterfactual campaigns, the daily rates of vaccination remained the same as at baseline. We focused specifically on the pediatric outcomes estimated by this model, which included isolation days, school absenteeism, and hospitalizations.

### Simulation Modeling Framework

We adapted our age-stratified agent-based model of COVID-19 to account for the waning of naturally acquired or vaccine-elicited immunity (eMethods in Supplement 1).^10,11,12,13,14,15^ The population was stratified into 10 age groups of 0 to 4, 5 to 10, 11 to 13, 14 to 17, 18 to 20, 21 to 29, 30 to 39, 40 to 49, and 50 to 64 years and 65 years or older based on US demographics.^16^ The model incorporated age-specific risk of hospitalizations, deaths, and contact patterns (eTable 1 and eMethods in Supplement 1).^17,18^ For the transmission dynamics, we considered the spread of 5 SARS-CoV-2 variants, including Iota, Alpha, Gamma, Delta, and Omicron, in addition to the original Wuhan-Hu-1 pandemic strain (eMethods in Supplement 1). Parameters of the transmission dynamics were based on the most recent estimates (eTable 2 in Supplement 1).

The model was calibrated by fitting to the reported incidence of COVID-19 between October 1, 2020, and February 28, 2022 (Figure and eMethods in Supplement 1).^7^ The fitted model was then simulated forward to March 31, 2023, under the baseline scenario and the 2 counterfactual scenarios of bivalent booster vaccination campaigns. Since the model calibration and fitting was based on the reported COVID-19 cases (and not the actual number of infections), the estimated outcomes corresponded to reported cases under the counterfactual scenarios.

**Figure.**
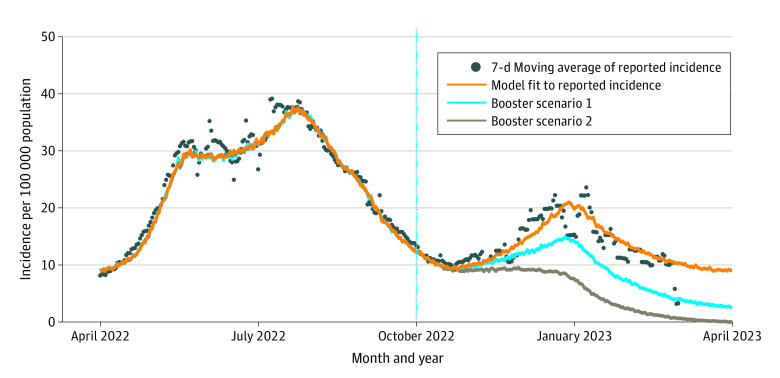
Model Fit Model fit to incidence with simulated scenarios of bivalent booster vaccination from October 1, 2022, to March 31, 2023. In booster scenario 1, booster uptake of eligible individuals reached one-half of the level of the age-specific influenza vaccination coverage in the 2020 to 2021 season. In booster scenario 2, booster uptake of eligible individuals reached the same level as the age-specific influenza vaccination coverage in the 2020 to 2021 season. Vertical dashed line indicates the start of these scenarios on October 1, 2022.

### Vaccination Scenarios Simulated

In counterfactual scenarios, the mean daily number of vaccine doses was maintained as the same value as the baseline value through September 30, 2022, before the start of accelerated booster vaccination campaigns. These campaigns were implemented between October 1 to December 31, 2022, during which the booster uptake of eligible individuals reached either one-half of the level (scenario 1) or the same level (scenario 2) as the age-specific influenza vaccination coverage in the 2020 to 2021 season. We considered the coverage in different age groups as the benchmark for booster campaigns. Benchmarks for scenario 2 were 59.0% for those aged 5 to 11 years, 51.0% for those aged 12 to 17 years, 38.0% for those aged 18 to 49 years, 54.0% for those aged 50 to 64 years, and 75.0% for those aged 65 years or older,^9^ with benchmarks for scenario 1 set at one-half of those levels (ie, 29.5% for ages 5-11 years, 25.5% for ages 12-17 years, 19.0% for ages 18-49 years, 27.0% for ages 50-64 years, and 37.5% for ages ≥65 years). As we expected diminishing marginal gains for increasing booster uptake when high coverage had already been achieved, the second counterfactual scenario provided an exploration of whether an ambitious booster campaign compared with the reported bivalent booster coverage of 23.3% of eligible individuals as of March 6, 2023,^19^ was worth the additional effort and resources.

Following the operational guidelines of the Centers for Disease Control and Prevention (CDC)^20^ and in light of authorization of pediatric bivalent boosters,^21^ we considered individuals aged 5 years or older to be eligible if at least 2 months had elapsed since their last dose of the primary series or a previous booster dose.

Vaccine effectiveness was specific to the regimen (primary vs booster), time since previous dose, and SARS-CoV-2 variant (eTables 3 and 4, eFigure 1, and eMethods in Supplement 1). Although the bivalent vaccines have been shown to generate significantly higher levels of neutralizing antibodies against the Omicron subvariants,^22,23^ estimates of their effectiveness in the general population are currently lacking. In our analysis, we set the effectiveness of a bivalent booster against infection, symptomatic disease, and severe disease caused by any Omicron subvariant to the corresponding estimates for a booster dose of the monovalent vaccines countering the BA.1 variant (eTables 3 and 4 and eMethods in Supplement 1).^24,25,26^

### Model Outcomes

We estimated the age-specific number of asymptomatic infections, mild symptomatic cases, severe nonhospitalized cases, hospitalized cases, and intensive care unit cases in the pediatric population aged 0 to 17 years across the study period of October 1, 2022, to March 31, 2023, for the baseline scenario and each counterfactual scenario. We further calculated isolation days among the entire pediatric population (aged 0 to 17 years) and school absenteeism among those aged 5 to 17 years. Guidance from the CDC stipulates that individuals who test positive for COVID-19 should isolate for at least 5 days and for at least 1 day beyond the resolution of symptoms.^27^ A minimum 10-day isolation period is advised for those who experience severe illness, including shortness of breath, low oxygen levels, or hospitalization. In our model, we calculated pediatric isolation days as 5 days per event for children who experienced mild symptomatic illness and 10 days per event for those with severe illness or hospitalization.

Considering that school is typically held only 5 days per week, when calculating days of school absenteeism, we multiplied pediatric isolation days among children aged 5 to 17 years by five-sevenths. We further adjusted the total days of absenteeism by 3.23% to account for children who received homeschooling.^28^

Based on the model estimates, we calculated the mean number of days of student and teacher absenteeism averted under each counterfactual scenario compared with the baseline scenario for typical school sizes at the elementary, middle, and high school levels. We obtained data on school enrollment and teacher count from the National Center for Education Statistics,^29^ stratified into tertiles as lower enrollment, middle enrollment, or higher enrollment, which we referred to as small, midsize, and large, respectively (Table 1). We assumed that students in elementary school were aged 5 to 10 years, students in middle school were aged 11 to 13 years, and students in high school were aged 14 to 17 years. We also assumed that teachers were representative of the population aged 21 to 64 years. To calculate student and teacher isolation days and absenteeism, we multiplied the estimated age-specific per capita isolation and absentee rates by the number of students or teachers at each category of school.

**Table 1.** Estimated Days of Student and Teacher Absenteeism Potentially Averted by Accelerated COVID-19 Bivalent Booster Vaccination Campaigns Between October 1, 2022, and March 31, 2023^a^

School level, individuals	Individuals enrolled, mean No.^b^	Mean (95% CrI), No.			
		Scenario 1		Scenario 2	
		Absent individuals	Days of absenteeism	Absent individuals	Days of absenteeism
**Elementary school**					
Small					
Students	214	5.5 (2.8-8.9)	21.5 (11.2-35.0)	10.4 (5.4-16.9)	40.7 (21.2-66.2)
Teachers	17	0.2 (0.1-0.3)	0.7 (0.4-1.1)	0.3 (0.2-0.5)	1.4 (0.8-2.1)
Midsize					
Students	412	4.8 (2.5-7.8)	18.9 (9.8-30.7)	9.4 (4.9-15.3)	35.4 (18.4-57.5)
Teachers	28	0.1 (0.1-0.2)	0.6 (0.4-0.9)	0.3 (0.2-0.5)	1.3 (0.8-1.9)
Large					
Students	669	6.4 (3.3-10.4)	25.1 (13.0-40.8)	11.3 (5.9-18.4)	44.5 (23.1-72.3)
Teachers	42	0.2 (0.1-0.3)	0.8 (0.5-1.2)	0.4 (0.2-0.5)	1.5 (0.9-2.3)
**Middle school**					
Small					
Students	233	7.8 (3.3-13.7)	30.7 (12.8-53.8)	14.6 (6.1-25.6)	57.5 (24.1-100.9)
Teachers	17	0.2 (0.1-0.4)	0.9 (0.4-1.5)	0.4 (0.2-0.7)	1.8 (0.8-2.9)
Midsize					
Students	556	6.8 (2.8-11.9)	27.0 (11.3-47.4)	13.3 (5.6-23.3)	52.2 (21.9-91.6)
Teachers	37	0.2 (0.1-0.3)	0.8 (0.4-1.3)	0.4 (0.2-0.6)	1.7 (0.8-2.7)
Large					
Students	976	9.1 (3.8-16.0)	35.7 (14.9-62.6)	16.1 (6.7-28.2)	62.9 (26.4-110.5)
Teachers	59	0.3 (0.1-0.4)	1.1 (0.5-1.7)	0.5 (0.2-0.8)	2.0 (0.9-3.2)
**High school**					
Small					
Students	110	6.3 (1.5-21.1)	24.6 (5.7-82.7)	11.9 (2.8-40.1)	47.0 (11.0-158.1)
Teachers	10	0.2 (0.1-0.5)	0.8 (0.3-2.3)	0.4 (0.1-1.0)	1.6 (0.5-4.5)
Midsize					
Students	47	5.5 (1.3-18.6)	21.5 (5.0-72.3)	10.9 (2.5-36.6)	42.8 (10.0-143.9)
Teachers	33	0.2 (0.1-0.5)	0.7 (0.2-2.0)	0.4 (0.1-1.0)	1.5 (0.5-4.1)
Large					
Students	1585	7.3 (1.7-24.6)	28.3 (6.6-95.3)	13.0 (3.0-43.9)	51.4 (12.0-172.9)
Teachers	90	0.2 (0.1-0.6)	1.0 (0.3-2.6)	0.4 (0.1-1.2)	1.8 (0.5-4.9)

Abbreviation: CrI, credible interval.

^a^ Accelerated bivalent booster coverage among eligible individuals was modeled using 2 counterfactual scenarios compared with the baseline scenario. In scenario 1, booster uptake was equal to one-half of the 2020 to 2021 age-specific influenza vaccination levels. In scenario 2, booster uptake was the same as the 2020 to 2021 age-specific influenza vaccination levels.

^b^ Data obtained from the National Center for Education Statistics.^29^

### Statistical Analysis

The simulation model was calibrated using 500 independent Monte Carlo realizations (eMethods in Supplement 1). For each Monte Carlo realization, we calculated the cumulative outcomes of simulations during the study period and derived 95% credible intervals (CrIs) using a bias-corrected and accelerated bootstrap method with 500 replications, which corrects for bias and skewness in the distribution of bootstrap estimates when scaled from the per capita population to the entire population. The adjustments for days of school absenteeism were applied to each independent Monte Carlo realization. The computational model was implemented in Julia language, and statistical analyses were conducted using Matlab software, version R2022B (MathWorks). For hypothesis testing, 2-sided *P* = .05 was considered to be significant.

## Results

Nationally, a COVID-19 bivalent booster campaign that achieved one-half of the age-specific coverage of the 2020 to 2021 influenza vaccination levels among eligible individuals was estimated to avert 4 506 119 (95% CrI, 3 956 423-5 208 525) isolation days among the pediatric population and 2 875 926 (95% CrI, 2 524 351-3 332 783) days of school absenteeism during the study period compared with the baseline scenario (Table 2). For the more ambitious bivalent booster campaign reaching the same level of influenza vaccination uptake for eligible individuals across all ages, the corresponding isolation and school absenteeism days averted were estimated to be 8 572 225 (95% CrI, 7 772 630-9 364 837) and 5 448 694 (95% CrI, 4 936 933-5 957 507), respectively.

**Table 2.** Estimates of Pediatric COVID-19 Outcomes Potentially Averted by Accelerated Vaccination Campaigns Between October 1, 2022, and March 31, 2023

Outcome^a^	Mean (95% CrI), No. averted^b^	
	Scenario 1	Scenario 2
Isolation days	4 506 119 (3 956 423-5 208 525)	8 572 225 (7 772 630-9 364 837)
Hospitalizations	5791 (4391-6932)	10 019 (8756-11 278)
Hospitalizations requiring ICU admission	1397 (846-1948)	2645 (2152-3147)
Days of school absenteeism	2 875 926 (2 524 351-3 332 783)	5 448 694 (4 936 933-5 957 507)

Abbreviations: CrI, credible interval; ICU, intensive care unit.

^a^ Averted isolation days, hospitalizations, and ICU admissions were estimated for the entire pediatric population aged 0 to 17 years. Averted days of school absenteeism were estimated for children aged 5 to 17 years.

^b^ Accelerated bivalent booster coverage among eligible individuals was modeled using 2 counterfactual scenarios compared with the baseline scenario. In scenario 1, booster uptake was equal to one-half of the 2020 to 2021 age-specific influenza vaccination levels. In scenario 2, booster uptake was the same as the 2020 to 2021 age-specific influenza vaccination levels.

To contextualize our results for local communities, we estimated the mean number of days of student and teacher absenteeism that could have been averted under each of the counterfactual scenarios compared with the baseline scenario for elementary, middle, and high schools of different sizes (Table 1). Specifically, the mean number of days of student absenteeism averted per school by a booster campaign that achieved one-half of the age-specific coverage of influenza vaccination ranged from 18.9 (95% CrI, 9.8-30.7) for midsize elementary schools to 25.1 (95% CrI, 13.0-40.8) for large elementary schools during the study period. For the same booster uptake scenario, the mean number of days of student absenteeism averted ranged from 27.0 (95% CrI, 11.3-47.4) for midsize middle schools to 35.7 (95% CrI, 14.9-62.6) for large middle schools and from 21.5 (95% CrI, 5.0-72.3) for midsize high schools to 28.3 (95% CrI, 6.6-95.3) for large high schools. As there were fewer teachers than students, the mean number of days of teacher absenteeism were substantially lower, ranging from 0.6 (95% CrI, 0.4-0.9) in midsize elementary schools to 1.1 (95% CrI, 0.5-1.7) in large middle schools. When the booster campaign mimicked the same uptake as age-specific influenza vaccination coverage, we found that the estimated mean number of days of student absenteeism averted was at least 75% higher for any school irrespective of its level or enrollment size.

For health outcomes, we estimated that a bivalent booster campaign achieving one-half of influenza vaccination–like coverage could have averted 5791 (95% CrI, 4391-6932) pediatric hospitalizations during the study period, of which 1397 (95% CrI, 846-1948) were estimated to require intensive care (Table 2). With booster coverage of eligible individuals reaching influenza vaccination–like uptake, we estimated that 10 019 (95% CrI, 8756-11 278) hospitalizations could have been averted, of which 2645 (95% CrI, 2152-3147) were estimated to require intensive care unit admission. From October 1, 2022, to March 4, 2023, more than 22 000 COVID-19 hospitalizations of children aged 0 to 17 years were reported.^19^ An influenza vaccination–like coverage of the COVID-19 bivalent booster could have reduced pediatric hospitalization by at least 45%.

We performed additional analyses assuming that only 50% of patients with mildly symptomatic cases followed guidelines for 5-day isolation (eFigure 2 in Supplement 1). The estimates of outcomes averted in each counterfactual scenario were similar to those obtained in the corresponding scenario when all patients with mildly symptomatic cases practiced 5-day isolation (eTable 5 in Supplement 1).

## Discussion

The findings of this decision analytical model highlighted the benefits that an accelerated COVID-19 bivalent booster campaign achieving high coverage across all age groups in the US could have provided for the pediatric population specifically. In addition to substantial reductions in pediatric illness and hospitalizations, our estimates revealed that widespread booster vaccination would have been associated with increased school attendance.

Reducing school absenteeism benefits students, families, and society as a whole. For students, reduced absenteeism maintains in-person learning, supporting educational recovery in the wake of pandemic-related learning loss.^4^ In addition to those who personally become ill, classroom outbreaks or teacher absenteeism may also be disruptive to students without infection. For families, school absenteeism may be associated with productivity losses and economic burden.^30^ In most cases, an adult caregiver would need to stay home with an isolated child. Our estimates of averted absenteeism do not include the benefits for parents, but isolation of children on weekend days may further strain parents who work on weekends. Fewer schedule disruptions among working parents and having well-educated and healthy children could prevent outcomes that have been found to be associated with reduced quality of life and socioeconomic burden.^31,32^

The reduction in adverse pediatric outcomes of COVID-19, especially hospitalization, is particularly salient during an academic year. The early onset and significant rise of both respiratory syncytial virus and influenza cases in autumn 2022 demonstrated how a concomitant surge of various respiratory diseases could strain pediatric hospital capacity nationwide.^33^ Improving bivalent booster uptake across all age groups may prevent recurrence of this scenario in future academic years as SARS-CoV-2 continues to evolve and become more transmissible and immune-evading variants emerge. Specifically, it is important to achieve high coverage in the pediatric population, as revealed when comparing the 2 counterfactual scenarios in our study. Indirect protection also plays a role and, in this decision analytical model, it was associated with benefits of booster vaccination for children younger than 5 years. Increasing booster coverage for the group aged 18 to 49 years from 19.0% in scenario 1 to 38.0% in scenario 2 was particularly important for indirect protection, as this age group includes many parents of school-aged children. Bivalent booster campaigns may emphasize the indirect benefits for children and school attendance to motivate this age group.

Achieving high booster uptake requires investment and outreach at the national level. Despite direct costs associated with vaccination (eg, vaccination clinic setup, vaccine storage and transportation, vaccine administration, vaccine doses, and vaccine waste) and those incurred indirectly as a result of work days lost due to vaccination clinic visits or adverse reactions to vaccines, the return on this investment could be substantial,^34^ with potential reductions in severe outcomes and school absenteeism.

### Limitations

Our analysis is subject to limitations. Specifically, we assumed that school absenteeism only applied to symptomatic children. The CDC guidance states that all individuals who test positive for COVID-19 should be isolated for at least 5 days irrespective of symptoms.^27^ Although testing of close contacts is far from universal, many people still test when they have a known infectious contact. This practice identifies a proportion of asymptomatic and presymptomatic cases in children, which may lead to additional absenteeism of students with infection. Even with reduced testing and increasing rates of asymptomatic infection and mildly symptomatic cases owing to the increase in immunity in the population and the propagation of less severe variants,^35,36^ the benefits of booster vaccination may be substantial (eTable 5 in Supplement 1). Our analysis assumed that the bivalent booster vaccines have the same effectiveness against the Omicron subvariants as those estimated for the monovalent booster vaccines against the Omicron BA.1 variant. Despite continual evolution of SARS-CoV-2 that challenges pandemic control, we did not consider the potential emergence of further immune-evasive variants in our analysis. Our model also did not include the use of COVID-19 outpatient treatments, as temporal data regarding uptake have been scarce. However, this omission is not likely to have substantial implications for pediatric outcomes, as treatment is most commonly directed to older adults.^37^

## Conclusions

In this decision analytical model, increased uptake of COVID-19 bivalent booster vaccination among eligible age groups was associated with decreased hospitalizations and school absenteeism in the pediatric population. These findings suggest that although COVID-19 prevention strategies often focus on older populations, the benefits of booster campaigns for children may be substantial, protecting them both directly and indirectly. In contrast, the cost of inaction may be high, with potentially millions more days of school absenteeism and thousands of preventable hospitalizations.

## Notes

Supplement 1. **eMethods.** Model Structure **eTable 1.** Mixing Patterns and the Daily Number of Contacts Derived From Empirical Observations **eTable 2.** Distribution of Disease Stages **eFigure 1.** Temporal Relative Effectiveness of Vaccines Against Infection and Severe Disease With 95% CIs Derived From a Gaussian Fit to Estimated Effectiveness After the Second Dose of Vaccines **eTable 3.** Estimated Vaccine Effectiveness (%) and Their 95% CIs From Published Studies for Monovalent Pfizer-BioNTech Vaccines **eTable 4.** Estimated Vaccine Effectiveness (%) and Their 95% CIs From Published Studies for Monovalent Moderna Vaccines **eFigure 2.** Model Fit to Incidence per 100 000 Population With Simulated Scenarios of Bivalent Booster Vaccination From October 1, 2022, to End of March 2023 **eTable 5.** Estimates of Averted Outcomes During the Study Period Between October 1, 2022, and March 31, 2023, Comparing Accelerated Booster Vaccination Campaigns With the Baseline Scenario When Only 50% of Mildly Symptomatic Cases Follow Isolation Guidelines
eReferences

Supplement 2.
Data Sharing Statement
