# Supplementary material for: Estimated US Pediatric Hospitalizations and School Absenteeism Associated With Accelerated COVID-19 Bivalent Booster Vaccination
Source: JAMA Netw Open. 2023 May 19;6(5):e2313586. doi: 10.1001/jamanetworkopen.2023.13586 (PMC10199352; doi:10.1001/jamanetworkopen.2023.13586)
Supplement: Supplement 1. — eMethods. Model Structure eTable 1. Mixing Patterns and the Daily Number of Contacts Derived From Empirical Observations eTable 2. Distribution of Disease Stages eFigure 1. Temporal Relative Effectiveness of Vaccines Against Infection and Severe Disease With 95% CIs Derived From a Gaussian Fit to Estimated Effectiveness After the Second Dose of Vaccines eTable 3. Estimated Vaccine Effectiveness (%) and Their 95% CIs From Published Studies for Monovalent Pfizer-BioNTech Vaccines eTable 4. Estimated Vaccine Effectiveness (%) and Their 95% CIs From Published Studies for Monovalent Moderna Vaccines eFigure 2. Model Fit to Incidence per 100 000 Population With Simulated Scenarios of Bivalent Booster Vaccination From October 1, 2022, to End of March 2023 eTable 5. Estimates of Averted Outcomes During the Study Period Between October 1, 2022, and March 31, 2023, Comparing Accelerated Booster Vaccination Campaigns With the Baseline Scenario When Only 50% of Mildly Symptomatic Cases Follow Isolation Guidelines eReferences [file jamanetwopen-e2313586-s001.pdf]

Supplementary Online Content

Fitzpatrick MC, Moghadas SM, Vilches TN, Shah A, Pandey A, Galvani AP. Estimated US pediatric hospitalizations and school absenteeism associated with accelerated COVID-19 bivalent booster vaccination. *JAMA Netw Open*. 2023;6(5):e2313586. doi:10.1001/jamanetworkopen.2023.13586

eMethods. Model Structure

eTable 1. Mixing Patterns and the Daily Number of Contacts Derived From Empirical Observations

eTable 2. Distribution of Disease Stages

eFigure 1. Temporal Relative Effectiveness of Vaccines Against Infection and Severe Disease With 95% CIs Derived From a Gaussian Fit to Estimated Effectiveness After the Second Dose of Vaccines

eTable 3. Estimated Vaccine Effectiveness (%) and Their 95% CIs From Published Studies for Monovalent Pfizer-BioNTech Vaccines

eTable 4. Estimated Vaccine Effectiveness (%) and Their 95% CIs From Published Studies for Monovalent Moderna Vaccines

eFigure 2. Model Fit to Incidence per 100 000 Population With Simulated Scenarios of Bivalent Booster Vaccination From October 1, 2022, to End of March 2023

eTable 5. Estimates of Averted Outcomes During the Study Period Between October 1, 2022, and March 31, 2023, Comparing Accelerated Booster Vaccination Campaigns With the Baseline Scenario When Only 50% of Mildly Symptomatic Cases Follow Isolation Guidelines

eReferences

This supplemental material has been provided by the authors to give readers additional information about their work.

© 2023 Fitzpatrick MC et al. *JAMA Network Open*.

eMethods. Model Structure

Model structure

We employed our previous agent-based model of COVID-19 transmission[1] and expanded its dynamic structure to account for waning of naturally-acquired or vaccine-elicited immunity, as well as booster vaccination. The model implemented natural history of COVID-19 with epidemiological classes of individuals as susceptible; latently infected (not yet infectious); asymptomatic (and infectious); pre-symptomatic (and infectious); symptomatic (and infectious) with either mild or severe illness; recovered; and dead. In addition, we considered individual-level timelines of eligibility for the primary series vaccination, booster doses with the monovalent vaccines, and booster doses with the bivalent vaccines.

The population was stratified into 10 age groups of 0 to 4, 5 to 10, 11-13, 14-17, 18 to 20, 21 to 29, 30 to 39, 40 to 49, 50 to 64, and 65+ years based on the US demographics,[2] and incorporated age-specific risk of hospitalizations and deaths,[3,4] as well as contact patterns. Daily contacts between individuals were sampled from age-specific negative-binomial distributions with parameters that accounted for the effect of interventions such as isolation of symptomatic cases (eTable 1).

© 2023 Fitzpatrick MC et al. *JAMA Network Open*.

eTable 1. Mixing Patterns and the Daily Number of Contacts Derived From Empirical Observations[5,6]

Daily numbers of contacts were sampled from negative binomial distributions for different scenarios.

Age group	Proportion of contacts between age groups					Mean no. of daily contacts (SD)	Mean no. of daily contacts for isolated individuals (SD)
	0-4	5-19	20-49	50-65	65+		
0-4	0.24	0.18	0.42	0.11	0.05	10.21 (7.65)	2.86 (2.14)
5-19	0.02	0.59	0.28	0.08	0.03	16.79 (11.72)	4.70 (3.28)
20-49	0.03	0.16	0.62	0.14	0.05	13.79 (10.50)	3.86 (2.95)
50-65	0.02	0.11	0.48	0.27	0.12	11.26 (9.59)	3.15 (2.66)
65+	0.02	0.11	0.40	0.22	0.25	8.00 (6.96)	2.24 (1.95)

SARS-CoV-2 variants

For the calibration and fitting the model to incidence data, we considered the spread of five variants, including Iota (B.1.526), Alpha (B.1.1.7), Gamma (P.1), Delta (B.1.617.2), and Omicron (B.1.1.529), in addition to the original Wuhan-Hu-I SARS-CoV-2 strain. All variants were introduced in the model at a date corresponding to twice the average duration of their estimated incubation period before the date of identification reported in the GISAID database.[7] Specifically, the Iota variant was introduced on October 25, 2020, with an estimated 35% higher transmissibility compared with the original Wuhan-Hu-I strain.[8] We then introduced the Alpha variant on November 29, 2020, with a 50% higher transmissibility compared to the original strain.[9,10] The Delta variant was inserted on February 10, 2021, with an elevated transmissibility of 30% compared with the Alpha variant.[11,12] Finally, we introduced the Omicron BA.1 variant on November 15, 2021, with a 35% higher transmissibility compared to Delta.[13]

Distribution of disease stages and infectiousness

The incubation period for each individual infected with a previous variant (Original, Iota, and Alpha) was sampled from a log-normal distribution with a mean of 5.2 days.[14] A proportion of infected individuals progressed to a pre-symptomatic stage[15,16] with an infectious period which was sampled from a Gamma distribution with a mean of 2.3 days.[15,17] The symptomatic disease following the pre-symptomatic stage had an average infectious period of 3.2 days, which was also sampled from a Gamma distribution.[15,17] The infectious period of individuals who remained asymptomatic was sampled from a Gamma distribution with a mean of 5 days.[15,18]

The incubation period for the Delta and Omicron variants were shorter [19–22] . For Delta, the incubation period was sampled from a lognormal distribution with a mean of 4.3 days, and a

© 2023 Fitzpatrick MC et al. *JAMA Network Open*.

pre-symptomatic duration of 2 days on average [20–22]. The incubation period for Omicron was also sampled from a log-normal distribution, but with a mean of 3.3 days[21]. The mean duration of the presymptomatic stage for Omicron was assumed to be the same as Delta with an average of 2 days.

Infectiousness was assumed to be highest during the pre-symptomatic stage. The transmissibilities during asymptomatic, mild symptomatic, and severe symptomatic stages were 26%, 44%, and 89%, respectively, relative to the pre-symptomatic stage.[17,23,24]

eTable 2. Distribution of Disease Stages

SARS-CoV-2 variants	Pre-symptomatic period	Incubation period	Infectious period	Transmissibility
Original strain	G(1.058, 5/2.3)	LogN(1.434, 0.661)	G(2.768, 1.1563)	β, Calibrated
Iota	G(1.058, 5/2.3)	LogN(1.434, 0.661)	G(2.768, 1.1563)	$1.35 \times \beta$
Alpha	G(1.058, 5/2.3)	LogN(1.434, 0.661)	G(2.768, 1.1563)	$1.50 \times \beta$
Gamma	G(1.058, 5/2.3)	LogN(1.434, 0.661)	G(2.768, 1.1563)	$1.60 \times \beta$
Delta	G(1.015, 1.975)	LogN(1.249, 0.649)	G(2.768, 1.1563)	$1.95 \times \beta$
Omicron	G(1.015, 1.975)	LogN(0.99, 0.64)	G(2.768, 1.1563)	$2.63 \times \beta$

LogN: Lognormal distribution

G: Gamma distribution

Disease outcomes

We assumed that asymptomatic and mild symptomatic individuals recover without hospitalization. Self-isolation was implemented to start within 24 hours of symptom onset for all symptomatic individuals, reducing their number of daily contacts by an average of 74% (eTable 1). Severely ill individuals due to primary infection were hospitalized within 2-5 days of symptom onset,[25,26] and therefore effectively excluded from the chain of disease transmission. The model was parameterized with rates of intensive care unit (ICU) and non-ICU admissions. [27–29] The risk of hospitalization with the Delta variant was assumed to be 2.26 times higher than that due to infection with Alpha.[29] We considered a 75.2% (95% confidence interval: 72.0% – 77.0%) risk reduction of hospitalization for severe disease due to infection by Omicron compared to Delta.[30,31] The risk of ICU admissions was reduced by 38.1% in severe patients of Omicron compared to those infected with Delta.[32]

Vaccination and immune dynamics

The number of vaccine doses per day and distribution of the first, second, and booster doses were parameterized with reported vaccination data in different age groups.[33] Following the start of vaccination, the booster eligibility was set to a 6-month period elapsed since the last dose of vaccine in fully vaccinated individuals. On January 3, 2022, this timeline was reduced to 5

© 2023 Fitzpatrick MC et al. *JAMA Network Open*.

months.[34] Given new guidelines, we simulated the model with a 4-month lag between the last vaccine dose or previous infection and a bivalent booster for eligible individuals.

We performed a literature review to derive the effectiveness estimates following each dose of the monovalent vaccines against infection, symptomatic disease, and severe disease for all variants in the model. Estimates of monovalent vaccine effectiveness for different SARS-CoV-2 variants in the model are summarized in eTables 3 and 4. We assume that the effectiveness of a bivalent booster against infection, symptomatic infection, and severe disease caused by more recent Omicron variants (BA.4/5, BQ.1, BQ.1.1) would be the same as the corresponding protection of a monovalent booster dose against the Omicron BA.1 variant.

To implement waning immunity after vaccination, we fitted a Gaussian model to estimates of vaccine effectiveness over time,[45–48] and determined the temporal relative effectiveness curves (eFigure 1). The relative effectiveness was then used as a multiplicative factor on the effectiveness of vaccines after the second or booster dose to determine the temporal immunity of individuals against infection and severe disease for each variant. We applied the same relative effectiveness for waning of naturally-acquired immunity. However, natural immunity was associated with 3.1 times (95% confidence interval: 1.4 – 4.8) lower risk of hospitalization compared to fully vaccinated individuals without a booster and no prior infection when Delta was the predominant variant.[49]

We considered primary, booster, and hybrid immunity in the model. Although hybrid immunity from infection plus two to three vaccine doses is shown to increase vaccine effectiveness with longer durability,[50,51] we did not have any specific quantification of such effectiveness for the duration of the study. We therefore conservatively assumed that vaccination of those with a previous infection will lead to the same protection estimated for the effectiveness of the second dose in the primary series or booster doses.

© 2023 Fitzpatrick MC et al. *JAMA Network Open*.

eFigure 1. Temporal Relative Effectiveness of Vaccines Against Infection and Severe Disease With 95% CIs Derived From a Gaussian Fit to Estimated Effectiveness After the Second Dose of Vaccines[45–47]

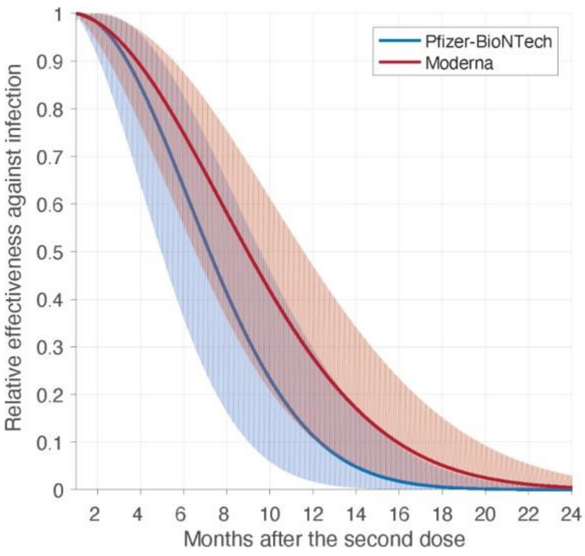

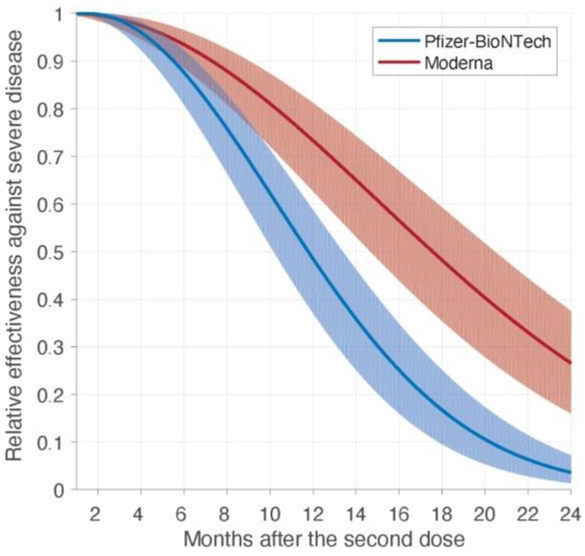

© 2023 Fitzpatrick MC et al. *JAMA Network Open*.

eTable 3. Estimated Vaccine Effectiveness (%) and Their 95% CIs From Published Studies for Monovalent Pfizer-BioNTech Vaccines

Booster dose restored or increased the protection effectiveness of two doses.

**Vaccine effectiveness (%)**	**Timelines**		**Reference**
Original strain and Iota variant	1 week after the second dose	1 week after the booster dose	35,36
Infection	86.1 (82.4, 89.1)	86.1 (82.4, 89.1)	
Symptomatic disease	93.0 (88.0, 95.0)	93.0 (88.0, 95.0)	
Severe disease	98.0 (90.0, 99.0)	98.0 (90.0, 99.0)	
Gamma variant			36,37
Infection	75.0 (70.5, 78.9)	75.0 (70.5, 78.9)	
Symptomatic disease	82.0 (65.0, 91.0)	82.0 (65.0, 91.0)	
Severe disease	96.0 (68.0, 99.0)	96.0 (68.0, 99.0)	
Alpha variant			36,37
Infection	89.5 (85.9, 92.3)	89.5 (85.9, 92.3)	
Symptomatic disease	89.0 (87.0, 90.0)	89.0 (87.0, 90.0)	
Severe disease	96.0 (94.0, 97.0)	96.0 (94.0, 97.0)	
Delta variant			36,38
Infection	85.0 (79.0, 90.0)	85.0 (79.0, 90.0)	
Symptomatic disease	92.0 (90.0, 94.0)	92.0 (90.0, 94.0)	
Severe disease	97.0 (96.0, 98.0)	97.0 (96.0, 98.0)	
Omicron variant			39,40
Infection	33.0 (31.0, 35.0)	76.0[a] (72.0, 79.0)	
Symptomatic disease	69 (62.0, 75.0)	82.0[a] (79.0, 84.0)	
Severe disease	81.0 (65.0, 90.0)	90.0[a] (80.0, 94.0)	

[a] Bivalent booster was assumed to have similar effectiveness.

© 2023 Fitzpatrick MC et al. *JAMA Network Open*.

eTable 4. Estimated Vaccine Effectiveness (%) and Their 95% CIs From Published Studies for Monovalent Moderna Vaccines

Booster dose restored or increased the protection effectiveness of two doses.

Vaccine effectiveness (%)	Timelines		Reference
Original strain and Iota variant	1 week after the second dose	1 week after the booster dose	36,41
Infection	96.4 (91.2, 98.5)	96.4 (91.2, 98.5)	
Symptomatic disease	96.0 (85.0, 99.0)	96.0 (85.0, 99.0)	
Severe disease	97.0 (78.0, 100.0)	97.0 (78.0, 100.0)	
Gamma variant			36,42
Infection	77.0 (63.0, 86.0)	77.0 (63.0, 86.0)	
Symptomatic disease	89.0 (21.0, 98.0)	89.0 (21.0, 98.0)	
Severe disease	95.0 (63.0, 99.0)	95.0 (63.0, 99.0)	
Alpha variant			36,41
Infection	98.4 (96.9, 99.1)	98.4 (96.9, 99.1)	
Symptomatic disease	92.0 (88.0, 95.0)	92.0 (88.0, 95.0)	
Severe disease	95.0 (92.0, 97.0)	95.0 (92.0, 97.0)	
Delta variant			36,43
Infection	86.7 (84.3, 88.7)	94.0 (92.3, 95.4)	
Symptomatic disease	95.0 (91.0, 97.0)	95.0 (91.0, 97.0)	
Severe disease	98.0 (93.0, 99.0)	98.0 (93.0, 99.0)	
Omicron variant			43,44
Infection	42.8 (33.8, 50.7)	67.7[b] (65.5, 69.7)	
Symptomatic disease	69.0 (62.0, 75.0)	82.0[b] (79.0, 84.0)	
Severe disease	81.0 (65.0, 90.0)	90.0[b] (80.0, 94.0)	

[b] Bivalent booster was assumed to have similar effectiveness

© 2023 Fitzpatrick MC et al. *JAMA Network Open*.

Model implementation

With the transmission probability derived from the calibration process, we fitted the model to incidence per 100,000 population from October 1, 2020 to September 30, 2022.[52] We chose October 1 as the starting point for our calibration and simulations because it was a time of a relatively low incidence preceding the fall/winter wave in the US, the launch of the vaccination campaign, and emergence of different variants. The pre-existing immunity against COVID-19 was included in the model using a probability distribution function, based on the reported incidence from the beginning of pandemic to the end of September 2020 to account for waning immunity over time (eFigure 1). At the start of fitting (October 2020), we assumed that contacts between individuals did not exceed 50% of the pre-pandemic level.[53] During the calibration process (in the presence of only the original strain of SARS-CoV-2), we determined the transmission probability of 0.0345 that minimized the difference between the mean of cumulative incidence predicted by running independent realizations in the model and cumulative reported cases over time. The transmission probability obtained during the calibration corresponds to an effective reproduction number of 1.17 in early October 2020.[54] After the calibration, the transmission probability of the original strain remained fixed, and the age- specific contact rates were adjusted to minimize the difference between the temporal cumulative incidence predicted by the model and the cumulative reported cases, implicitly accounting for the change and effect of various non-pharmaceutical measures. During fitting, the transmissibility of other variants was adjusted relative to the preceding variant(s) at the time they were introduced in the model. We then ran 500 independent Monte-Carlo simulations for the study period, and determined the 95% credible intervals using a bias-corrected and accelerated bootstrap method (with 500 replications), which corrects for bias and skewness in the distribution of bootstrap estimates when scaled from the per capita to the entire US population. The model was implemented in Julia, and simulation codes are available at:
https://github.com/thomasvilches/USomicron/tree/booster_scenarios

Additional results

We simulated the model when only 50% of mildly symptomatic cases follow guidelines for isolation. eFigure 2 illustrates the model projection for the incidence per 100,000 population with simulated scenarios of bivalent booster vaccination from October 1, 2022 to end of March 2023.

© 2023 Fitzpatrick MC et al. *JAMA Network Open*.

eFigure 2. Model Fit to Incidence per 100 000 Population With Simulated Scenarios of Bivalent Booster Vaccination From October 1, 2022, to End of March 2023

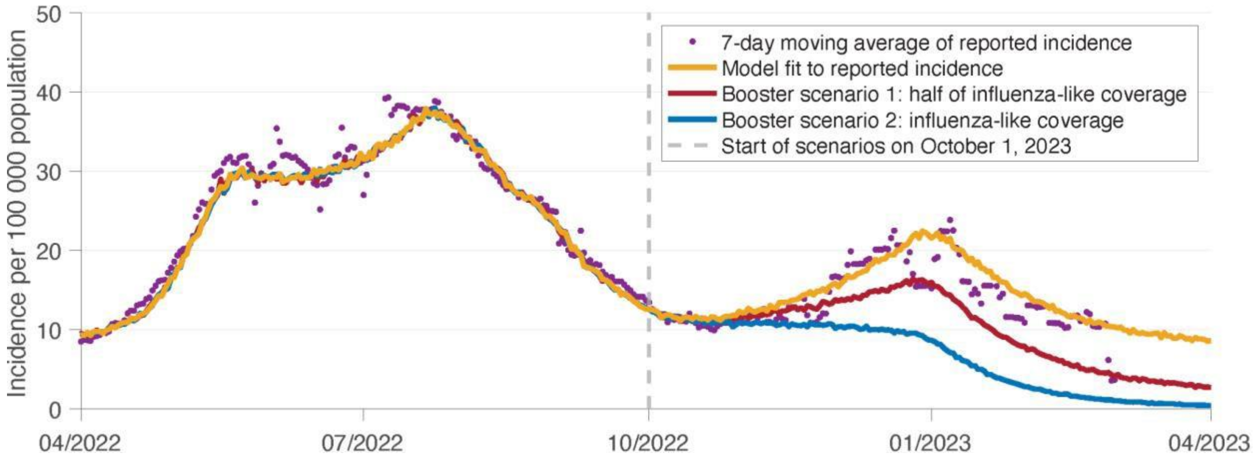

© 2023 Fitzpatrick MC et al. *JAMA Network Open*.

eTable 5. Estimates of Averted Outcomes During the Study Period Between October 1, 2022, and March 31, 2023, Comparing Accelerated Booster Vaccination Campaigns With the Baseline Scenario When Only 50% of Mildly Symptomatic Cases Follow Isolation Guidelines

Averted outcomes for isolation days, hospitalizations, and ICU hospitalizations were estimated for the entire pediatric population aged 0-17 years. Averted days of school absenteeism were estimated for children aged 5-17 years. Accelerated bivalent booster coverage among eligible individuals were: (Scenario 1) equal to half of the 2020–21 age-specific influenza vaccination levels; and (Scenario 2) equal to the 2020–21 age-specific influenza vaccination levels.

**Averted COVID-19 outcomes: mean (95% credible interval)**		
**Outcome**	**Scenario 1**	**Scenario 2**
Isolation days	5,071,120<br>(4,396,166 - 5,849,385)	9,149,378<br>(8,200,998 - 9,992,184)
Hospitalizations	5,953<br>(4,533 - 7,153)	10,954<br>(9,533 - 12,362)
Hospitalizations requiring ICU	1,047<br>(232 - 1,808)	2,225<br>(1,569 - 2,912)
Days of school absenteeism[1]	3,183,120<br>(2,765,796 - 3,642,786)	5,692,221<br>(5,123,495 - 6,215,083)

[1] Among children aged 5-17.

© 2023 Fitzpatrick MC et al. *JAMA Network Open*.

eReferences

1. Vilches TN, Moghadas SM, Sah P, et al. Estimating COVID-19 Infections, Hospitalizations, and Deaths Following the US Vaccination Campaigns During the Pandemic. *JAMA Netw Open*. 2022;5(1):e2142725.

2. Explore census data. Accessed January 25, 2022. https://data.census.gov/cedsci/table?q=United%20States&table=DP05&tid=ACSDP1Y2017.DP05&g=0100000US&lastDisplayedRow=29&vintage=2017&layer=state&cid=DP05_0001 E

3. Moghadas SM, Vilches TN, Zhang K, et al. The Impact of Vaccination on Coronavirus Disease 2019 (COVID-19) Outbreaks in the United States. *Clin Infect Dis*. Published online January 30, 2021. doi:10.1093/cid/ciab079

4. CDC. Risk for COVID-19 Infection, Hospitalization, and Death By Age Group. Centers for Disease Control and Prevention. Published November 8, 2022. Accessed December 26, 2022. https://www.cdc.gov/coronavirus/2019-ncov/covid-data/investigations-discovery/hospitalization-death-by-age.html

5. Mossong J, Hens N, Jit M, et al. Social contacts and mixing patterns relevant to the spread of infectious diseases. *PLoS Med*. 2008;5(3):e74.

6. Jarvis CI, Van Zandvoort K, Gimma A, et al. Quantifying the impact of physical distance measures on the transmission of COVID-19 in the UK. *BMC Med*. 2020;18(1):124.

7. Outbreak.Info. outbreak.info. Accessed March 28, 2022. https://outbreak.info/location-reports?loc=USA&dark=true

8. Annavajhala MK, Mohri H, Wang P, et al. Emergence and Expansion of the SARS-CoV-2 Variant B.1.526 Identified in New York. *medRxiv*. Published online August 4, 2021. doi:10.1101/2021.02.23.21252259

9. Davies NG, Abbott S, Barnard RC, et al. Estimated transmissibility and impact of SARS-CoV-2 lineage B.1.1.7 in England. *Science*. 2021;372(6538). doi:10.1126/science.abg3055

10. Davies NG, Jarvis CI, CMMID COVID-19 Working Group, et al. Increased mortality in community-tested cases of SARS-CoV-2 lineage B.1.1.7. *Nature*. Published online March 15, 2021. doi:10.1038/s41586-021-03426-1

11. Allen H, Vusirikala A, Flannagan J, et al. Household transmission of COVID-19 cases associated with SARS-CoV-2 delta variant (B.1.617.2): national case-control study. *Lancet Reg Health Eur*. 2022;12:100252.

12. Dhar MS, Marwal R, Vs R, et al. Genomic characterization and epidemiology of an emerging SARS-CoV-2 variant in Delhi, India. *Science*. 2021;374(6570):995-999.

13. Yang W, Shaman J. SARS-CoV-2 transmission dynamics in South Africa and epidemiological characteristics of the Omicron variant. *medRxiv*. Published online December 21, 2021. doi:10.1101/2021.12.19.21268073

14. Li Q, Guan X, Wu P, et al. Early Transmission Dynamics in Wuhan, China, of Novel

© 2023 Fitzpatrick MC et al. *JAMA Network Open*.

Coronavirus–Infected Pneumonia. *N Engl J Med*. 2020;382(13):1199-1207.

15. Li R, Pei S, Chen B, et al. Substantial undocumented infection facilitates the rapid dissemination of novel coronavirus (SARS-CoV-2). *Science*. 2020;368(6490):489-493.

16. Sah P, Fitzpatrick MC, Zimmer CF, et al. Asymptomatic SARS-CoV-2 infection: A systematic review and meta-analysis. *Proc Natl Acad Sci U S A*. 2021;118(34). doi:10.1073/pnas.2109229118

17. Moghadas SM, Fitzpatrick MC, Sah P, et al. The implications of silent transmission for the control of COVID-19 outbreaks. *Proc Natl Acad Sci U S A*. 2020;117(30):17513-17515.

18. Gatto M, Bertuzzo E, Mari L, et al. Spread and dynamics of the COVID-19 epidemic in Italy: Effects of emergency containment measures. *Proc Natl Acad Sci U S A*. 2020;117(19):10484-10491.

19. Kang M, Xin H, Yuan J, et al. Transmission dynamics and epidemiological characteristics of Delta variant infections in China. *medRxiv*. Published online August 13, 2021:2021.08.12.21261991. doi:10.1101/2021.08.12.21261991

20. Xin H, Wong JY, Murphy C, et al. The Incubation Period Distribution of Coronavirus Disease 2019: A Systematic Review and Meta-analysis. *Clin Infect Dis*. 2021;73(12):2344-2352.

21. Jansen L, Tegomoh B, Lange K, et al. Investigation of a SARS-CoV-2 B.1.1.529 (Omicron) Variant Cluster - Nebraska, November-December 2021. *MMWR Morb Mortal Wkly Rep*. 2021;70(5152):1782-1784.

22. Grant R, Charmet T, Schaeffer L, et al. Impact of SARS-CoV-2 Delta variant on incubation, transmission settings and vaccine effectiveness: Results from a nationwide case-control study in France. *The Lancet Regional Health - Europe*. 2022;13:100278. doi:10.1016/j.lanepe.2021.100278

23. Ferretti L, Wymant C, Kendall M, et al. Quantifying SARS-CoV-2 transmission suggests epidemic control with digital contact tracing. *Science*. 2020;368(6491). doi:10.1126/science.abb6936

24. Sayampanathan AA, Heng CS, Pin PH, Pang J, Leong TY, Lee VJ. Infectivity of asymptomatic versus symptomatic COVID-19. *Lancet*. 2021;397(10269):93-94.

25. Shoukat A, Wells CR, Langley JM, Singer BH, Galvani AP, Moghadas SM. Projecting demand for critical care beds during COVID-19 outbreaks in Canada. *CMAJ*. 2020;192(19):E489-E496.

26. Moghadas SM, Shoukat A, Fitzpatrick MC, et al. Projecting hospital utilization during the COVID-19 outbreaks in the United States. *Proc Natl Acad Sci U S A*. 2020;117(16):9122-9126.

27. Garg S, Kim L, Whitaker M, et al. Hospitalization Rates and Characteristics of Patients Hospitalized with Laboratory-Confirmed Coronavirus Disease 2019 - COVID-NET, 14 States, March 1-30, 2020. *MMWR Morb Mortal Wkly Rep*. 2020;69(15):458-464.

28. Team CC 19 R, CDC COVID-19 Response Team, Chow N, et al. Preliminary Estimates of

© 2023 Fitzpatrick MC et al. *JAMA Network Open*.

the Prevalence of Selected Underlying Health Conditions Among Patients with Coronavirus Disease 2019 — United States, February 12–March 28, 2020. *MMWR Morbidity and Mortality Weekly Report*. 2020;69(13):382-386. doi:10.15585/mmwr.mm6913e2

29. Twohig KA, Nyberg T, Zaidi A, et al. Hospital admission and emergency care attendance risk for SARS-CoV-2 delta (B.1.617.2) compared with alpha (B.1.1.7) variants of concern: a cohort study. *Lancet Infect Dis*. Published online August 27, 2021. doi:10.1016/S1473-3099(21)00475-8

30. Scribner H. Omicron variant leads to less severe symptoms, deaths, new study says. *Deseret news (Salt Lake City, Utah: 1964)*. https://www.deseret.com/coronavirus/2021/12/31/22861222/omicron-variant-less-severe-covid-symptoms-deaths. Published December 31, 2021. Accessed January 6, 2022.

31. UK Health Security Agency. *Technical Briefing: Update on Hospitalisation and Vaccine Effectiveness for Omicron VOC-21NOV-01 (B.1.1.529).*; 2021. https://assets.publishing.service.gov.uk/government/uploads/system/uploads/attachment_data/file/1045619/Technical-Briefing-31-Dec-2021-Omicron_severity_update.pdf

32. Maslo C, Friedland R, Toubkin M, Laubscher A, Akaloo T, Kama B. Characteristics and Outcomes of Hospitalized Patients in South Africa During the COVID-19 Omicron Wave Compared With Previous Waves. *JAMA*. Published online December 30, 2021. doi:10.1001/jama.2021.24868

33. CDC. COVID data tracker. Centers for Disease Control and Prevention. Published March 28, 2020. Accessed December 16, 2021. https://covid.cdc.gov/covid-data-tracker/

34. Office of the Commissioner. Coronavirus (COVID-19) update: FDA takes multiple actions to expand use of Pfizer-BioNTech COVID-19 vaccine. U.S. Food and Drug Administration. Published January 3, 2022. Accessed January 6, 2022. https://www.fda.gov/news-events/press-announcements/coronavirus-covid-19-update-fda-takes-multiple-actions-expand-use-pfizer-biontech-covid-19-vaccine

35. Dagan N, Barda N, Kepten E, et al. BNT162b2 mRNA Covid-19 Vaccine in a Nationwide Mass Vaccination Setting. *N Engl J Med*. 2021;384(15):1412-1423.

36. Nasreen S, Chung H, He S, et al. Effectiveness of COVID-19 vaccines against symptomatic SARS-CoV-2 infection and severe outcomes with variants of concern in Ontario. *Nat Microbiol*. Published online February 7, 2022. doi:10.1038/s41564-021-01053-0

37. Abu-Raddad LJ, Chemaitelly H, Butt AA, National Study Group for COVID-19 Vaccination. Effectiveness of the BNT162b2 Covid-19 Vaccine against the B.1.1.7 and B.1.351 Variants. *N Engl J Med*. Published online May 5, 2021. doi:10.1056/NEJMc2104974

38. Pouwels KB, Pritchard E, Matthews PC, et al. Effect of Delta variant on viral burden and vaccine effectiveness against new SARS-CoV-2 infections in the UK. *Nat Med*. 2021;27(12):2127-2135.

39. Andeweg SP, de Gier B, Eggink D, et al. Protection of COVID-19 vaccination and previous infection against Omicron BA.1 and Delta SARS-CoV-2 infections, the Netherlands, 22 November 2021- 19 January 2022. *bioRxiv*. Published online February 7, 2022. doi:10.1101/2022.02.06.22270457

© 2023 Fitzpatrick MC et al. *JAMA Network Open*.

40. Ferdinands JM. Waning 2-Dose and 3-Dose Effectiveness of mRNA Vaccines Against COVID-19–Associated Emergency Department and Urgent Care Encounters and Hospitalizations Among Adults During Periods of Delta and Omicron Variant Predominance — VISION Network, 10 States, August 2021–January 2022. *MMWR Morb Mortal Wkly Rep*. 2022;71. doi:10.15585/mmwr.mm7107e2

41. Bruxvoort KJ, Sy LS, Qian L, et al. Effectiveness of mRNA-1273 against delta, mu, and other emerging variants of SARS-CoV-2: test negative case-control study. *BMJ*. 2021;375:e068848.

42. Charmet T, Schaeffer L, Grant R, et al. Impact of original, B.1.1.7, and B.1.351/P.1 SARS-CoV-2 lineages on vaccine effectiveness of two doses of COVID-19 mRNA vaccines: Results from a nationwide case-control study in France. *Lancet Reg Health Eur*. 2021;8:100171.

43. Tseng HF, Ackerson BK, Luo Y, et al. Effectiveness of mRNA-1273 against SARS-CoV-2 omicron and delta variants. *bioRxiv*. Published online January 8, 2022. doi:10.1101/2022.01.07.22268919

44. Thompson MG, Natarajan K, Irving SA, et al. Effectiveness of a Third Dose of mRNA Vaccines Against COVID-19-Associated Emergency Department and Urgent Care Encounters and Hospitalizations Among Adults During Periods of Delta and Omicron Variant Predominance - VISION Network, 10 States, August 2021-January 2022. *MMWR Morb Mortal Wkly Rep*. 2022;71(4):139-145.

45. Cohn BA, Cirillo PM, Murphy CC, Krigbaum NY, Wallace AW. SARS-CoV-2 vaccine protection and deaths among US veterans during 2021. *Science*. Published online November 4, 2021:eabm0620.

46. Tartof SY, Slezak JM, Fischer H, et al. Effectiveness of mRNA BNT162b2 COVID-19 vaccine up to 6 months in a large integrated health system in the USA: a retrospective cohort study. *Lancet*. 2021;398(10309):1407-1416.

47. Chemaitelly H, Yassine HM, Benslimane FM, et al. mRNA-1273 COVID-19 vaccine effectiveness against the B.1.1.7 and B.1.351 variants and severe COVID-19 disease in Qatar. *Nature Medicine*. 2021;27(9):1614-1621. doi:10.1038/s41591-021-01446-y

48. Wright BJ, Tideman S, Diaz GA, French T, Parsons GT, Robicsek A. Comparative vaccine effectiveness against severe COVID-19 over time in US hospital administrative data: a case-control study. *Lancet Respir Med*. Published online February 25, 2022. doi:10.1016/S2213-2600(22)00042-X

49. León TM, Dorabawila V, Nelson L, et al. COVID-19 Cases and Hospitalizations by COVID-19 Vaccination Status and Previous COVID-19 Diagnosis - California and New York, May-November 2021. *MMWR Morb Mortal Wkly Rep*. 2022;71(4):125-131.

50. Altarawneh HN, Chemaitelly H, Ayoub HH, et al. Effects of Previous Infection and Vaccination on Symptomatic Omicron Infections. *N Engl J Med*. 2022;387(1):21-34.

51. Carazo S, Skowronski DM, Brisson M, et al. Protection against omicron (B.1.1.529) BA.2 reinfection conferred by primary omicron BA.1 or pre-omicron SARS-CoV-2 infection among health-care workers with and without mRNA vaccination: a test-negative case-control study. *Lancet Infect Dis*. 2023;23(1):45-55.

© 2023 Fitzpatrick MC et al. *JAMA Network Open*.

52. *Covid-19-Data: An Ongoing Repository of Data on Coronavirus Cases and Deaths in the U.S*. Github Accessed December 23, 2022. https://github.com/nytimes/covid-19-data

53. Schlosser F. Contact Behavior in the Pandemic. Published November 3, 2021. Accessed December 6, 2021. https://www.covid-19-mobility.org/reports/contacts-pandemic/

54. Krieger M, Owens T. *Rt COVID-19*. Accessed May 2, 2021. https://github.com/rtcovidlive/

© 2023 Fitzpatrick MC et al. *JAMA Network Open*.
