# Supplementary material for: Estimated US Pediatric Hospitalizations and School Absenteeism Associated With Accelerated COVID-19 Bivalent Booster Vaccination
Source: JAMA Netw Open. 2023 May 19;6(5):e2313586. doi: 10.1001/jamanetworkopen.2023.13586 (PMC10199352; doi:10.1001/jamanetworkopen.2023.13586)
Supplement: Supplement 2. — Data Sharing Statement [file jamanetwopen-e2313586-s002.pdf]

Data Sharing Statement

Fitzpatrick. Estimated US Pediatric Hospitalizations and School Absenteeism Associated With Accelerated COVID-19 Bivalent Booster Vaccination. *JAMA Netw Open*. Published May 19, 2023. doi:10.1001/jamanetworkopen.2023.13586

Data
Data available: Yes
Data types: Other (please specify)
Additional Information: All data used in the study are from publicly available sources and are referred in the manuscript. All data and results generated by simulation model would be publicly available on a public github page.
How to access data: https://github.com/thomasvilches/USomicron/tree/booster_scenarios
When available: With publication

Supporting Documents
Document types: Statistical/analytic code
How to access documents: It can be accessed from github repository: https://github.com/thomasvilches/USomicron/tree/booster_scenarios
When available: With publication

Additional Information
Who can access the data: Anyone
Types of analyses: Any purpose
Mechanisms of data availability: Publicly available
